# Comparison of Surface Integrity and Fatigue Performance on TC17 Titanium Alloy After Shot Peening and Re-Shot Peening

**DOI:** 10.3390/ma19102090

**Published:** 2026-05-16

**Authors:** Chengsong Liu, Pengfei Ma, Pengfei Liu, Dan Liu, Jiale Guo

**Affiliations:** 1 School of 3D Printing, Xinxiang University, Xinxiang 453003, China; hnnumapengfei@sina.cn (P.M.); flashsky1234@sina.com (P.L.);; 2 National & Local Joint Engineering Laboratory for High-Performance Metal Structure Materials and Advanced Manufacturing Technology, Guizhou University, Guiyang 550025, China; liud@gzu.edu.cn

**Keywords:** surface integrity, fatigue performance, compressive residual stress, shot peening, re-shot peening

## Abstract

**Highlights:**

- High-intensity peening causes surface damage and surface compressive residual stress relaxation.
- RSP smooths indentations, raises compressive residual stress, and deepens the affected layer.
- At 750 MPa, RSP improves fatigue life by 45.9-fold compared with the BM specimen.
- Optimal fatigue performance requires balancing beneficial and detrimental factors.
- Surface compressive residual stress is the primary determinant of fatigue enhancement.

**Abstract:**

In this study, we have systematically investigated how both shot peening (SP) and re-shot peening (RSP) affect the surface integrity and fatigue performance of TC17 titanium alloy. Surface integrity involves a competitive mechanism with the coexistence of beneficial and detrimental factors, both of which collectively determine the fatigue performance. While compressive residual stress (CRS), dislocation multiplication, and work hardening serve as beneficial factors, surface roughness and surface damage act as the detrimental factors. The results reveal high-intensity SP produced a deeper work-hardened layer. However, it also caused larger surface roughness and more severe surface damage, yielding the relaxation of the surface CRS and poorer improvement of the fatigue performance than that achieved with medium-intensity peening. The RSP treatment demonstrated a superior balance of surface integrity factors by effectively reducing the degree of surface damage and elevating the magnitude of surface CRS. Thus, a 45.9-fold improvement in fatigue life was achieved relative to the base material (BM) specimen. Surface CRS was identified as the primary factor contributing to the enhanced fatigue performance.

## 1. Introduction

Due to its superior integration of high strength, excellent fracture toughness, good hardenability, and wide forgeability temperature range, the typical dual-phase (α + β) TC17 titanium alloy is commonly utilized to manufacture components, including fans, compressor disks, and centrifugal impellers [1,2,3]. However, titanium alloys usually have some inherent drawbacks—low hardness, poor wear resistance, and high notch sensitivity. Furthermore, these components also experience complex cyclic loading in service, making their fatigue performance critical to the safety and reliability of aero-engines. Typically, the nucleation of fatigue cracks occurs at component surfaces, where the tensile stresses are greatest. Extensive investigations have indicated that surface integrity exerts a critical influence on the fatigue performance of metallic materials [3,4,5,6,7,8,9,10,11,12]. Shot peening (SP) is a conventional, effective, and low-cost treatment, which essentially modifies the surface integrity by a series of plastic deformation processes.

Surface integrity after SP treatment can generally be summarized as the following four factors: compressive residual stress (CRS), work hardening, microstructure (e.g., dislocation multiplication, grain refinement, and nanocrystalline structure), and surface quality (e.g., surface roughness and surface damage) [5,6,13,14,15]. While CRS, work hardening, and microstructural refinement can effectively suppress fatigue crack initiation and early propagation [8,16,17], excessive surface roughness and surface damage promote stress concentration and thereby deteriorate fatigue performance. Hence, SP treatment is commonly employed to improve the fatigue performance of metallic materials [8,12,14,17,18,19,20]. For example, Sheng et al. [12] observed that SP introduced CRS and grain refinement in TiAl alloy, enhancing its high-cycle fatigue life (at 800 MPa) by 280%. Trško et al. [14] revealed that SP induced a nanocrystalline structure and a deep CRS in the surface layer of 50CrMo4 steel, with a consequent increase in its very high cycle fatigue strength (at 10^9^ cycles) by almost 23%. Wang et al. [19] noted that cryogenic SP induced a higher dislocation density, grain refinement, and CRS in the surface layer of 2024-T351 aluminum alloy, which improved its fatigue life (at 310 MPa, R = 0.06) by up to 2.85 times compared to the untreated specimen.

These competing surface effects are inherently interdependent. For example, overly high peening intensity often has a double-edged effect [5,20,21,22]. On the one hand, it is likely to induce a more pronounced strengthening effect; on the other hand, the process is also likely to create significant surface defects, including excessive surface roughness, microcracks, folding, and even delamination [6,9,18]. Such defects tend to create stress concentration and promote CRS relaxation during cyclic loading, restricting the enhancement of fatigue performance [23]. González et al. [5] indicated that for Al6063 alloy, severe SP induced a fine-grained layer, but it also made surface roughness much worse. As a result, fatigue strength improved by only 16%. By contrast, conventional SP with milder parameters raised it by 28%. As reported by Maleki et al. [6], excessively high-intensity SP induced microcracks on the AISI 1050 steel surface, lowering the fatigue limit by 6.1%. However, upon removal of the damaged layer, there was a marked enhancement in fatigue performance, evidenced by an increase of more than 44.4% in the fatigue limit. Calvo-García et al. [18] also illustrated that excessively high-intensity peening on AZ31B magnesium alloy led to microcracking and increased the surface roughness from 0.41 μm to 1.79 μm, which significantly weakened the enhancement of fatigue performance by SP. Thus, the key to enhancing fatigue performance by SP is to achieve a proper balance between beneficial and detrimental factors, and a reasonable range of peening intensity must be ensured. Excessively high-intensity peening increases surface roughness and generates surface damage, resulting in a significant weakening of the fatigue strengthening effect [18,23]. Although low-intensity peening yields a relatively good surface quality, it introduces a lower level of CRS, making it difficult to realize the desired fatigue strengthening effect [12].

To better balance the beneficial and detrimental factors, a re-shot peening (RSP) treatment was proposed, in which a re-peening process is carried out after the initial SP treatment with a lower intensity. Through this treatment, the beneficial factors introduced by the initial peening stage, such as the CRS, the fine-grained microstructure, and the work-hardened layer, can be preserved [6,9]. Surface microcracks are repaired or closed by the re-peening step, which in turn enhances the surface quality [6,9]. The same conclusions have been reached by Bagherifard et al. [13]. Although SP treatment generated a nanocrystalline structure and a deep CRS layer, the accompanying severe surface damage restricted the enhancement of fatigue strength to only 4%. By smoothing the surface damage, RSP treatment gave a 10% enhancement in fatigue strength.

For TC17 titanium alloy, a critical material for aerospace structural applications, surface integrity can directly influence its fatigue performance. Several works have documented the influence of SP on the surface integrity and fatigue performance of TC17 titanium alloy [3,24,25]. As an example, Tan et al. [3] noted that the surface integrity of TC17 titanium alloy was markedly enhanced by SP treatment, yielding peak surface CRS and surface microhardness. This shifted the fatigue crack nucleation sites to the subsurface layers 56–100 μm below the surface, increasing the fatigue life by 92% compared to the milling condition. Shi et al. [24] reported that SP combined with vibration finishing enhanced the room-temperature fatigue performance of TC17 titanium alloy more than SP treatment. When subjected to 300 °C for 100 h, the specimen still maintained a high-temperature fatigue performance 18.2% higher than that of the substrate, due to CRS, gradient microstructure, and reduced surface roughness. Yu et al. [25] noted that multiple SP (three passes) of TC17 titanium alloy generated a thicker nanocrystalline layer and higher CRS relative to conventional SP, while also markedly reducing surface roughness. Despite these findings, a clear consensus on which specific factors dictate the fatigue enhancement of this alloy is still lacking. Furthermore, compared with SP, the influence of RSP on the surface integrity and fatigue performance of TC17 titanium alloy remains insufficiently explored.

To fill the above research gaps, this study systematically investigates the influence of SP and RSP on the surface integrity and fatigue performance of TC17 titanium alloy. Unlike previous studies that mainly evaluated the combined influence of surface integrity factors, this study employs factor-separation experiments to distinguish the roles of surface roughness and surface CRS in fatigue performance. Through the analysis of microstructure, surface quality, microhardness, residual stress, and fatigue performance of this alloy under different peening conditions, the competitive and cooperative roles of beneficial and detrimental factors are elucidated. Ultimately, the results offer practical engineering guidance and quantitative insights for optimizing the peening processes for TC17 titanium alloy aerospace components.

## 2. Materials and Methods

### 2.1. TC17 Titanium Alloy

**Figure 1:**
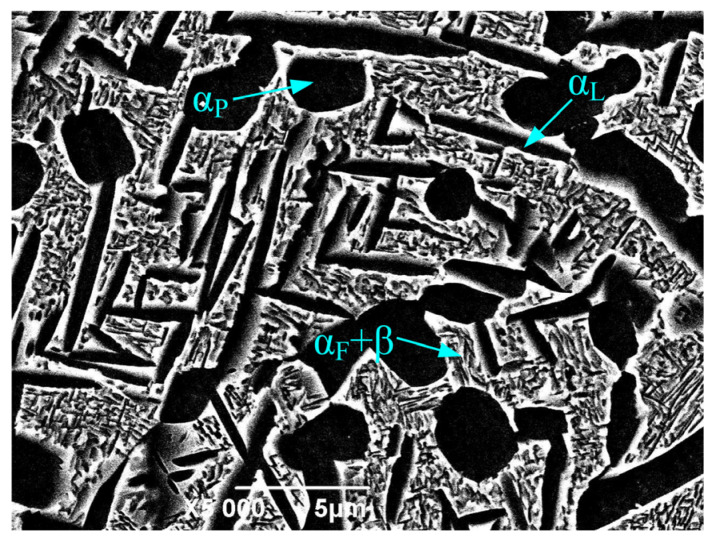
Microstructure of TC17 titanium alloy.

The experimental material was TC17 titanium alloy (BaoTi Group Ltd., Baoji, China) in the form of annealed bar stock. This alloy was solution-treated at 840 °C (1 h) with water quenching, then aged at 640 °C (8 h) with air cooling. After heat treatment, the microstructure was composed of equiaxed primary α grains (α_P_, diameter: 1–4 μm), along with lamellar secondary α (α_L_), fine acicular secondary α (α_F_), and intergranular β phases, as shown in Figure 1. The corresponding alloy contains the following chemical composition (mass %): 5.1Al, 4.1Mo, 4.0Cr, 2.38Sn, 2.0Zr, 0.03Fe, 0.01N, 0.01C, 0.001H, 0.07O, with the balance being Ti. The alloy exhibits a yield strength of 1163 MPa, a tensile strength of 1193 MPa, an elongation of 14%, and a reduction in area of 44.7%.

### 2.2. Preparation of Fatigue Specimens

An air blast SP device (8558 Type, Qingdao Spancer Machinery Co., Ltd., Qingdao, China) was utilized for applying SP and RSP treatments to the fatigue test specimens. To examine how Almen intensity affects surface integrity and fatigue performance, three peening intensity levels (0.25, 0.35, and 0.45 mmN) were adopted using S110 cast steel shot, with all other parameters held constant. Z300 ceramic shots were chosen for the re-peening process due to their ability to produce a superior surface finish. Table 1 lists the peening parameters along with corresponding specimen IDs.

### 2.3. Fatigue Tests

Information on the configuration and dimensional parameters of the fatigue specimens is provided in [26]. Fatigue tests were performed using a PQ-6 rotary bending apparatus (Ningxia Qing Shan Testing Machine Co. Ltd., Yinchuan, China) at 50 Hz (R = −1). All tests were conducted at ambient temperature, with at least three parallel specimens tested at each stress level.

### 2.4. Measurement Methods and Apparatus

To examine the surface microstructure, transmission electron microscopy (TEM) investigation was carried out on a field-emission instrument (Tecnai G2 F20, FEI Company, Hillsboro, OR, USA) set to 200 kV. Specimen preparation for TEM observation was conducted as follows: (1) a thin sheet measuring approximately 12 × 12 × 0.8 mm was sectioned from the surface; (2) the sheet was mechanically ground starting from the inner face until its thickness reached roughly 50 μm; (3) final thinning was performed by twin-jet electropolishing using a mixed solution of 10% HClO_4_ and 90% CH_3_OH until perforation. Phase identification was carried out via an X-ray diffractometer (XRD, D/max 2500, Rigaku, Tokyo, Japan) employing Cu-Kα radiation. The instrument was operated at 40 kV and 30 mA, with a scanning step of 0.02° and a 2θ scanning range from 30° to 80°. A scanning electron microscope (SEM, JSM-6390, JEOL Ltd., Tokyo, Japan) was utilized to examine the two-dimensional (2D) surface morphology and fracture characteristics. A confocal laser scanning microscope (C130, Lasertec Corporation, Yokohama, Japan) was employed to explore the three-dimensional (3D) surface topography and surface roughness. Microhardness profiles were obtained with a microhardness tester (MHV-1000, Sinowon Ltd., Dongguan, China) using a Knoop indenter (load: 25 g, holding time: 20 s). At each depth, three parallel measurements were averaged to obtain the reported hardness value. To determine the stress state, X-ray diffraction measurements were undertaken on an Xstress 3000 diffractometer (Stresstech Oy, Vaajakoski, Finland). The sin^2^Ψ approach was applied on the {213} plane of α-Ti (2θ: 134–148°, irradiated area: 3.14 mm^2^). To assess the residual stress variation with depth, consecutive thin layers on the surface were etched away using a mixed solution of HNO_3_ and HF, after which X-ray diffraction measurements were performed.

## 3. Results

### 3.1. Microstructure

**Figure 2:**
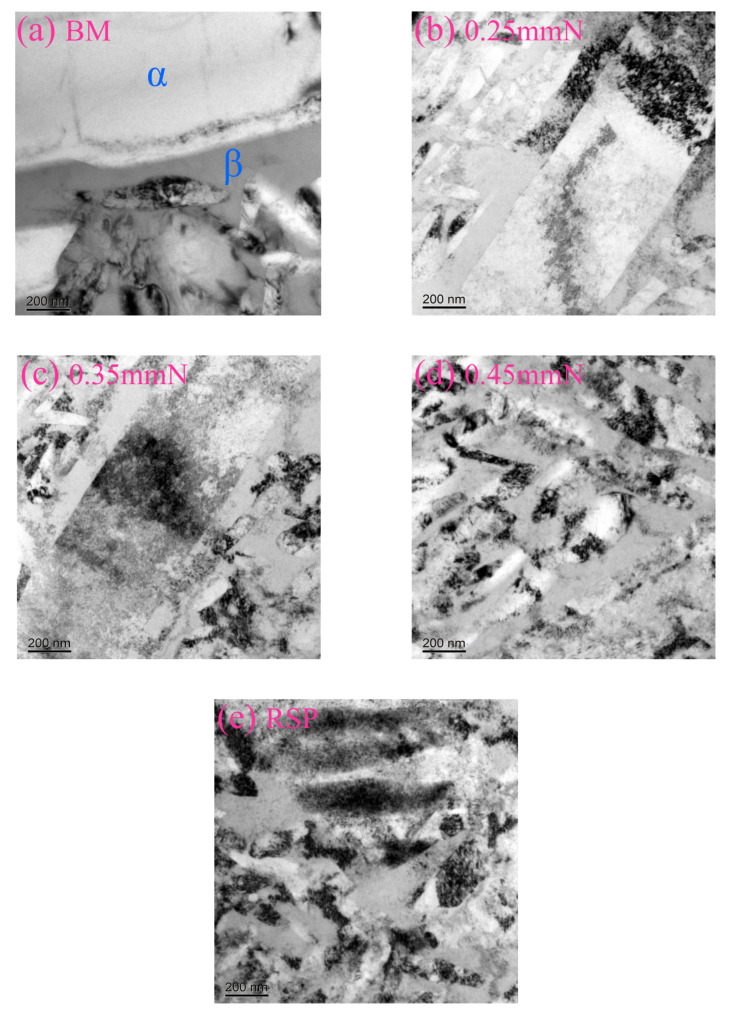
TEM images from the near-surface regions of: (**a**) BM specimen (low dislocation density); (**b**) 0.25 mmN specimen (dislocation tangles and cells); (**c**) 0.35 mmN specimen (dense dislocation walls and tangles); (**d**) 0.45 mmN specimen (extremely high dislocation density); (**e**) RSP specimen (densest dislocation structures, fine cells and walls). Here, α represents the alpha phase, and β represents the beta phase.

Figure 2 provides the TEM images from the near-surface regions of the base material (BM) and the specimens after various SP treatments. Only a few dislocations are present in the BM specimen, exhibiting a low dislocation density (Figure 2a). Following SP and RSP treatments, the material surface underwent significant plastic deformation, which led to dislocation slip and substantial multiplication (Figure 2b–e). As dislocations continuously multiplied and slipped, they accumulated to form a high density of dislocations, including dislocation tangles, cells, and walls. For the SP-treated specimens, the dislocation density increased with peening intensity. A particularly dense dislocation structure was observed in the 0.35 mmN and 0.45 mmN specimens (Figure 2c,d). After RSP treatment, the dislocation tangles became denser and more dislocation structures were formed (Figure 2e). This elevation of dislocation density improved the yield strength, indicating that a higher applied stress was needed to sustain further deformation. As a result, fatigue crack initiation and propagation were effectively hindered, which constitutes a microstructural mechanism underlying the improved fatigue performance [7].

**Figure 3:**
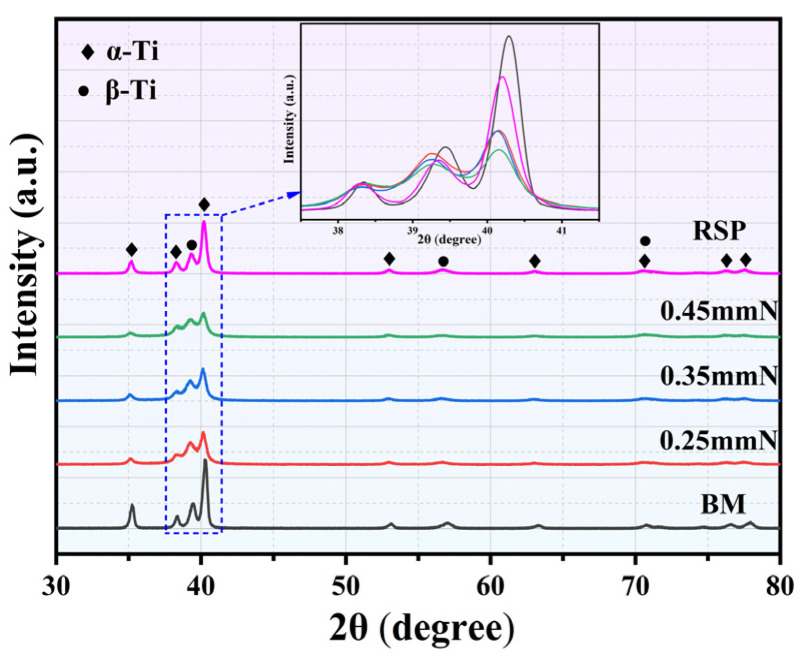
XRD patterns obtained from the BM, 0.25 mmN, 0.35 mmN, 0.45 mmN, and RSP specimens.

XRD patterns obtained from the BM, SP-, and RSP-treated specimens are illustrated in Figure 3. It is evident that for both the SP- and RSP-treated specimens, the Bragg diffraction peaks were broadened and shifted. For titanium alloys with high stacking fault energy, plastic deformation is dominated by dislocation slip, with the consequent lattice distortion mainly manifested as dislocation formation and grain refinement [27]. The observed peak broadening and shifting thus arise from the synergistic interplay between grain refinement and microstrain [16,20].

### 3.2. Surface Topography and Surface Roughness

**Figure 4:**
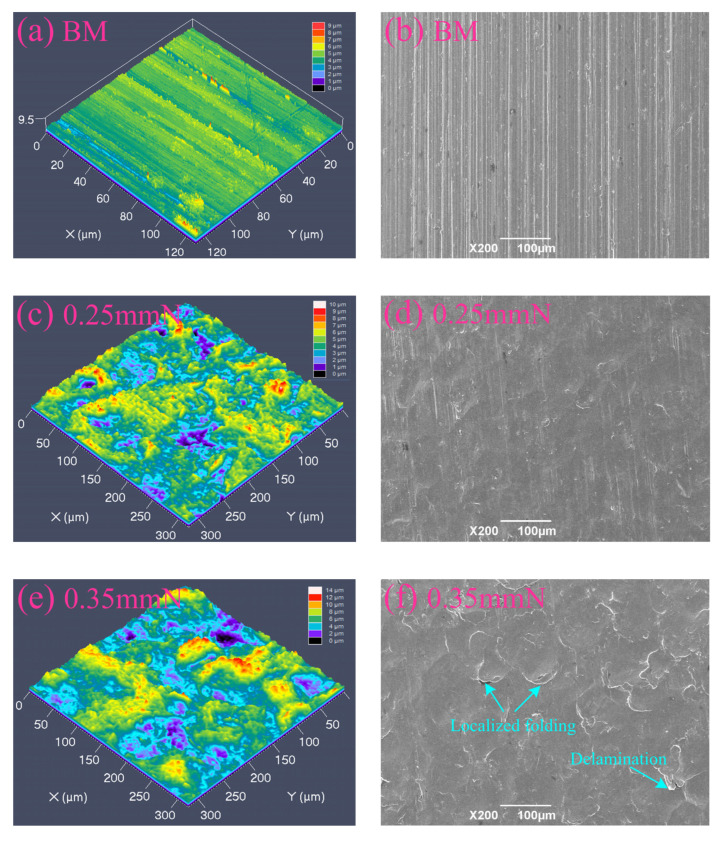

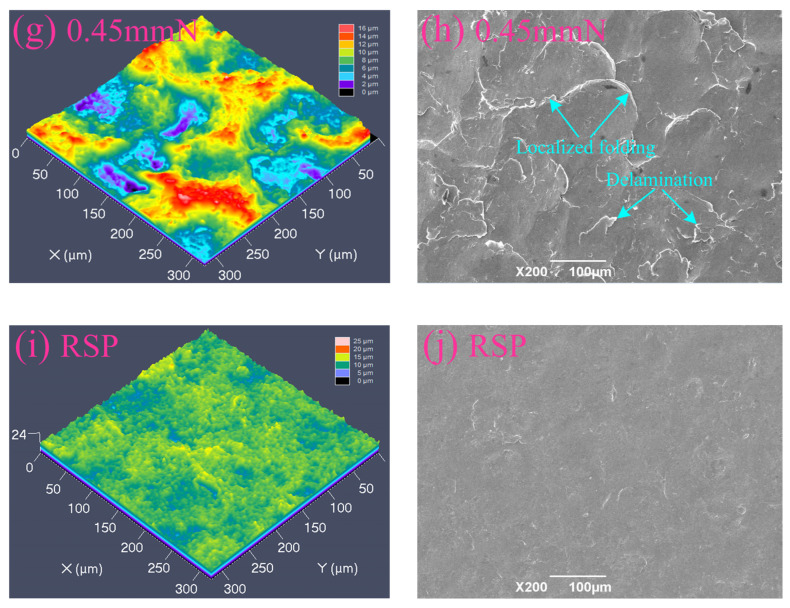
Comparison of the 3D surface topography and 2D surface morphology: (**a**,**b**) BM, (**c**,**d**) 0.25 mmN, (**e**,**f**) 0.35 mmN, (**g**,**h**) 0.45 mmN, and (**i**,**j**) RSP specimens.

**Figure 5:**
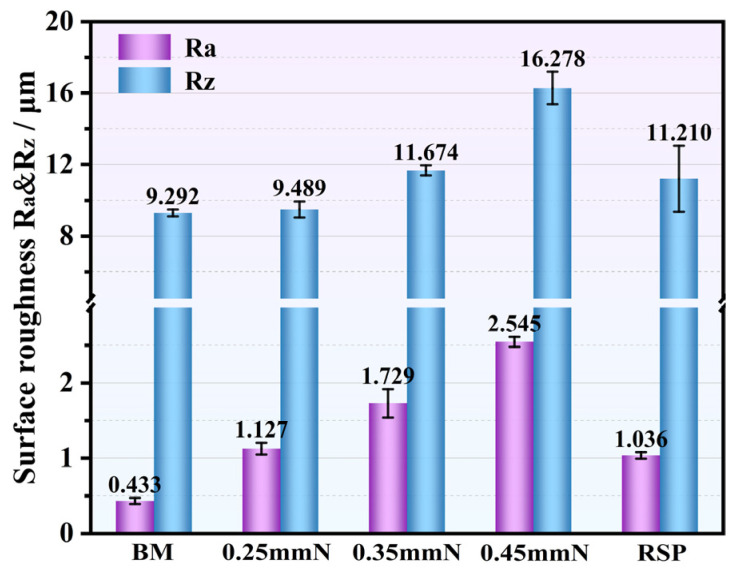
Surface roughness values measured on the BM, 0.25 mmN, 0.35 mmN, 0.45 mmN, and RSP specimens.

A comparative assessment of the 3D topography and 2D morphology for the BM, SP-, and RSP-treated specimens is displayed in Figure 4, and their corresponding surface roughness values are summarized in Figure 5. As shown in Figure 4a,b, the BM specimen surface retains distinct mechanical polishing marks, featuring an Ra value of 0.433 μm and an Rz value of 9.292 μm (Figure 5). By contrast, all SP- and RSP-treated specimens exhibit prominent peening indentations and a typical undulated topography, leading to significantly higher Ra and Rz values than those of the BM condition. In the case of the 0.25 mmN specimen, the surface was characterized by numerous shallow peening indentations, while localized material folding remained negligible (Figure 4c,d). With increasing peening intensity up to 0.35 mmN, the peening indentations grew in both dimensions and depth. The edges of several peening indentations revealed localized folding and delamination (Figure 4e,f). With a further increase in peening intensity, the surface of the 0.45 mmN specimen exhibited significant surface damage, with extensive localized folding and delamination at the edges of the peening indentations (Figure 4g,h). With a rise in peening intensity, the material surface underwent increasingly severe plastic deformation, which drove a steady escalation of surface roughness. This progressive increase in surface damage yielded Ra values for the 0.25 mmN, 0.35 mmN, and 0.45 mmN specimens that were, respectively, approximately 1.6, 3.0, and 4.9 times greater relative to the BM specimen (Figure 5). In contrast, the deep peening indentations and surface damage (localized folding and delamination), formed by the initial peening were effectively smoothed out by the RSP treatment (Figure 4i,j). A key effect of RSP treatment is that plastic deformation promotes near-surface material flow, which reduces surface asperities and alleviates stress concentration at surface defect sites. As a result, the surface roughness was reduced, with the Ra value falling to approximately 40.1% lower than that of the 0.35 mmN specimen (Figure 5).

### 3.3. Microhardness

**Figure 6:**
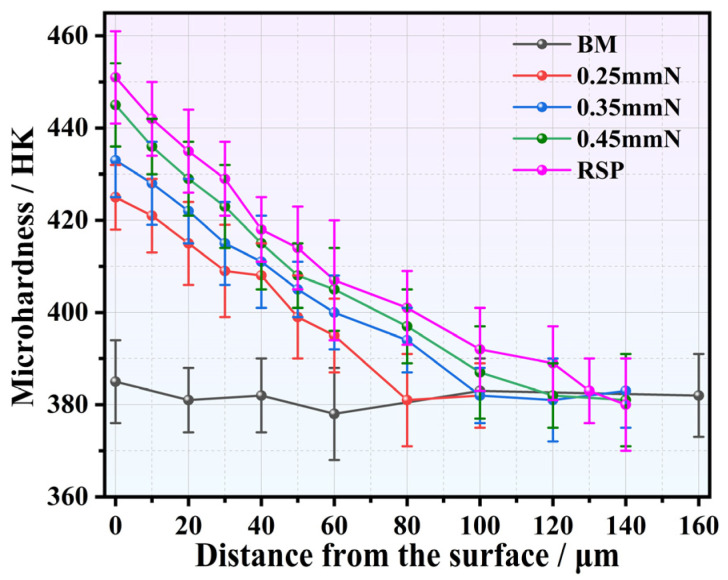
Depth-dependent microhardness distributions of the BM, SP-, and RSP-treated specimens.

Figure 6 compares depth-dependent microhardness distributions of the BM, SP-, and RSP-treated specimens. A hardness of approximately 380 HK was measured for the BM specimen. For specimens subjected to SP treatments at varying intensities, the near-surface microhardness was significantly elevated, exhibiting a characteristic gradient profile that peaked near the outermost surface and then progressively decayed with increasing depth toward the BM. For the specimens SP-treated at 0.25, 0.35, and 0.45 mmN, the surface microhardness reached 425, 433, and 445 HK, while the work-hardened layers extended to roughly 80, 100, and 120 μm, respectively.

Relative to the initial peening of the 0.35 mmN specimen, the RSP treatment further improved the surface microhardness to 451 HK and extended the work-hardened layer to roughly 130 μm. These improvements in hardness can be attributed to a high-density dislocation structure formed at the surface for the SP- and RSP-treated conditions. A clear trend can also be observed: the higher the dislocation density, the higher the surface microhardness. Such a work-hardened layer can effectively impede fatigue crack initiation [10,11,19].

### 3.4. Residual Stress

**Figure 7:**
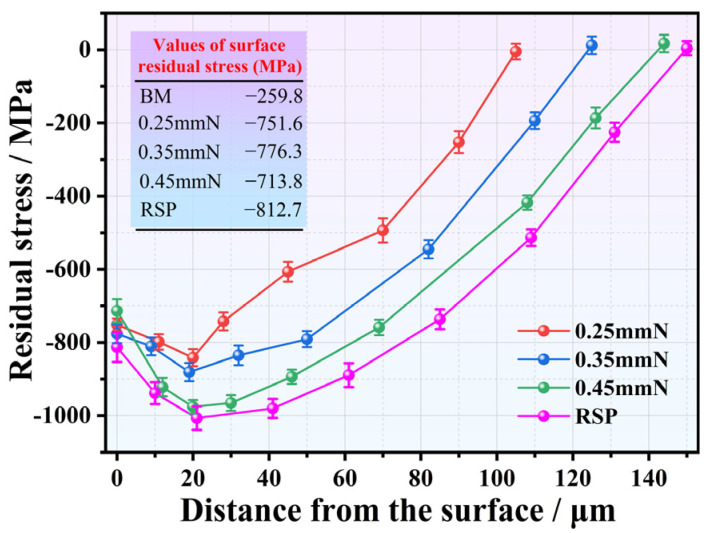
Residual stress distributions for the SP- and RSP-treated specimens.

A comparison of the residual stress distributions for the SP- and RSP-treated conditions is displayed in Figure 7. Following machining and polishing, the BM specimen showed a surface CRS of 259.8 MPa. After SP treatment with different Almen intensities, significant CRS layers were produced, with corresponding affected depths varying from roughly 105 to 145 μm. It can also be found that the affected depth of CRS tends to increase with rising peening intensity.

The values of surface CRS were 751.6 and 776.3 MPa for the 0.25 mmN and 0.35 mmN specimens, respectively. As peening intensity increased to 0.45 mmN, the value instead decreased to 713.8 MPa. Such a non-monotonic trend primarily stems from the evolution of the surface morphology. From Figure 5, a gradual increase in surface roughness can be seen with elevated peening intensity. When the peening intensity reached 0.45 mmN, severe surface damage characterized by local folding and delamination was observed. Such surface damage induced obvious stress concentration, which relaxed the surface CRS and thus weakened the fatigue strengthening effect [4,21,28].

For all SP- and RSP-treated specimens, residual stress distributions exhibited a typical hook-shaped pattern, in which the maximum CRS values of 841–1005 MPa occurred at roughly 20 μm beneath the surface. The existence of CRS restrains fatigue crack nucleation and initial propagation, thus significantly enhancing fatigue resistance [12,16,19]. Moreover, the RSP treatment served to further optimize the stress distribution. Compared with the 0.35 mmN specimen, RSP not only increased the surface CRS from 776.3 to 812.7 MPa but also extended the affected depth from approximately 124 to 150 μm.

### 3.5. Fatigue Life and S-N Curves

**Figure 8:**
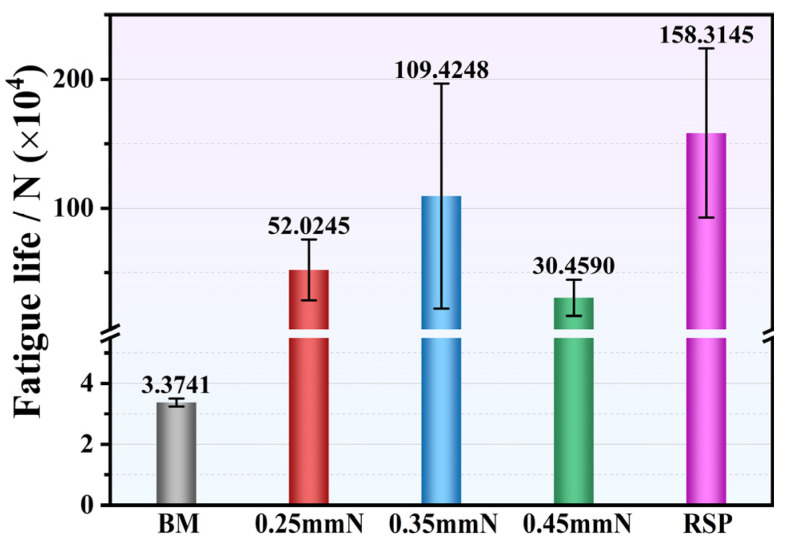
Fatigue life results for the BM, SP-, and RSP-treated specimens (maximum stress: 750 MPa).

From three parallel tests, the fatigue life results for the BM, SP-, and RSP-treated specimens (maximum stress: 750 MPa) are presented in Figure 8. Compared to the BM specimen, all SP treatments significantly improved fatigue life, with increases of 14.4, 31.4, and 8.0 times for 0.25, 0.35, and 0.45 mmN specimens, respectively. With an increase in peening intensity from 0.25 to 0.35 mmN, the fatigue life improved markedly. For the 0.35 mmN specimen, a higher surface CRS (776.3 MPa), a deeper work-hardened layer (about 124 μm), and milder surface damage (Ra: 1.729 μm) were obtained, leading to an optimized balance between beneficial and detrimental factors. With a further increase to 0.45 mmN, however, the fatigue life declined significantly. Under this peening condition, although the affected depths of the work-hardened (Figure 6) and CRS (Figure 7) were further extended, this also led to a sharp rise in surface roughness (Figure 5) and severe surface damage (Figure 4h). As such, the expected further enhancement of fatigue performance was not attained. The detrimental factors, such as the very rough surface and surface damage, eventually yielded a shorter fatigue life compared with the 0.35 mmN-treated specimen.

The RSP treatment produced the greatest improvement in fatigue life, increasing it by 45.9-fold relative to the BM specimen. This remarkable improvement can be attributed to the collaborative optimization of surface integrity obtained by RSP treatment. The re-peening treatment effectively smoothed the initial peening indentations and mitigated surface damage (Figure 4i,j), resulting in a 40.1% reduction in Ra value. Moreover, the re-peening treatment further enhanced the affected depth of CRS from 124 μm (0.35 mmN specimen) to 150 μm (RSP specimen). The corresponding value of surface CRS also elevated from 776.3 to 812.7 MPa. The enhanced fatigue performance of RSP specimens arises from promoted beneficial effects and weakened detrimental ones.

**Figure 9:**
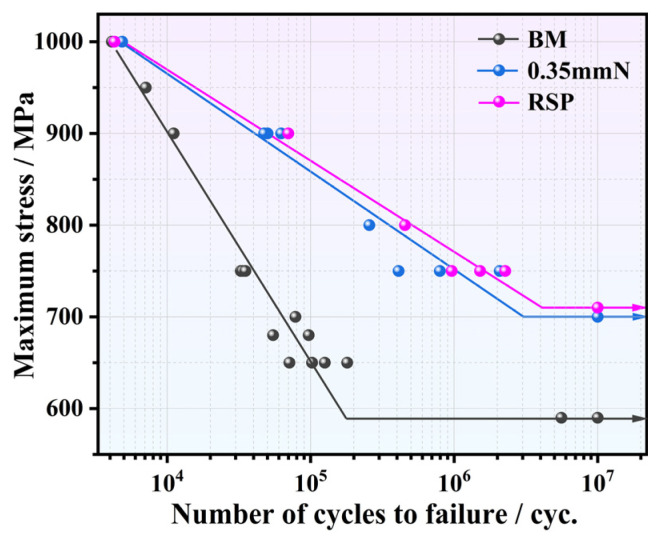
S-N curves of the BM, 0.35 mmN, and RSP specimens.

Figure 9 displays the S-N curves, which correlate maximum stress with cycles to failure, obtained from the BM, 0.35 mmN, and RSP specimens. The BM specimen yielded a fatigue limit of approximately 590 MPa (at 10^7^ cycles). After SP and RSP treatment, these fatigue limits were remarkably enhanced relative to the BM specimen. The 0.35 mmN specimen exhibited an 18.6% improvement in fatigue limit from 590 MPa (BM condition) to 700 MPa. In contrast, the RSP treatment yielded further gains, achieving a fatigue limit of 710 MPa, which corresponds to increases of 20.3% over the BM specimen and 1.4% over the 0.35 mmN specimen. It further reveals that the improvement in fatigue performance induced by the SP and RSP treatments is more pronounced under low stress conditions than under high stress conditions. This arises primarily from the lower susceptibility of the induced surface CRS to relaxation under reduced stress amplitudes [29].

### 3.6. Fractography Observations

**Figure 10:**
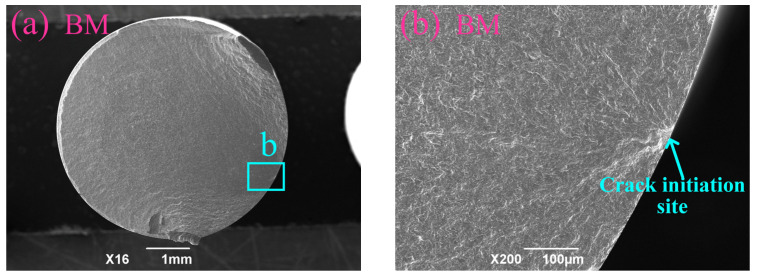

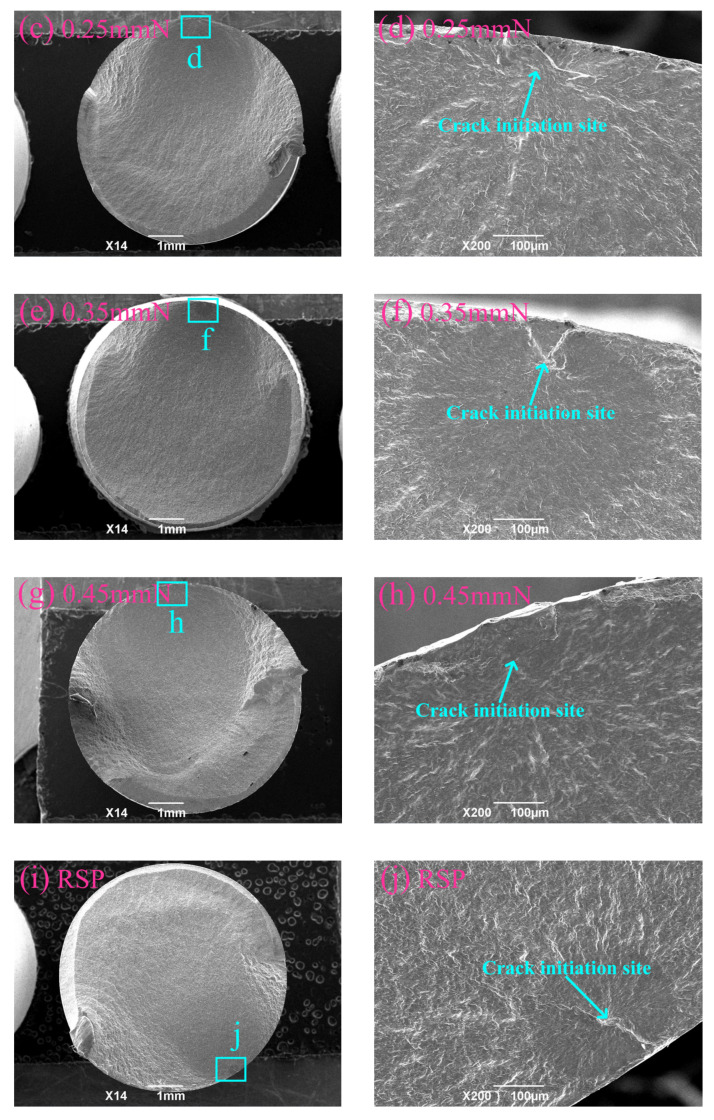
Fracture surface morphology of the (**a**,**b**) BM, (**c**,**d**) 0.25 mmN, (**e**,**f**) 0.35 mmN, (**g**,**h**) 0.45 mmN, and (**i**,**j**) RSP specimens.

Figure 10 presents the fatigue fracture surface morphology corresponding to the specimens in Figure 8. A single crack initiation site was observed for each specimen. The BM specimen exhibited the characteristic of surface fatigue crack nucleation, whereas for the SP- and RSP-treated specimens, initiation occurred in the subsurface, roughly 40–60 μm beneath the surface. The CRS induced by SP partly offsets the applied cyclic loading, causing a relocation of the maximum effective fatigue stress to the subsurface [8]. Meanwhile, the work hardening generated in the near-surface region by SP further contributed to suppressing the crack nucleation. The subsurface shift in fatigue cracks indicates that surface crack nucleation is effectively hindered, suggesting improved fatigue performance [7,10,12,30].

## 4. Discussion

As can be observed in Figure 8, SP treatments at various peening intensities (0.25, 0.35, and 0.45 mmN) improved the fatigue performance to varying degrees. Especially, the 0.35 mmN specimen exhibited the most pronounced enhancement in fatigue performance. However, at a higher intensity of 0.45 mmN, the fatigue life decreased, falling below that of the 0.35 mmN specimen. A similar trend can be observed in the literature [4,18,28,30,31]. This phenomenon highlights the trade-off effect of SP on surface integrity. Specifically, neither too low nor too high a peening intensity yields the optimal strengthening effect.

Mechanistically, the enhanced fatigue performance stems from combined effects of beneficial factors—including CRS, dislocation multiplication, and work hardening. One contributing factor is the CRS from SP, which partially offsets the applied alternating load, inhibits fatigue crack initiation and early propagation, and consequently improves fatigue performance [24,32]. Another factor is the high-density dislocation tangles, cells, and walls induced by SP within the surface layer (Figure 2b–d). Through this microstructural evolution, a substantial work-hardening effect is achieved, which effectively suppresses fatigue crack initiation [12,20,23,30,33]. Both CRS and work-hardening contribute to enhanced fatigue performance, but their primary role is to suppress crack initiation rather than to retard crack propagation [3,8,12,23]. However, the SP treatment inevitably brought about some detrimental effects, including elevated surface roughness (Figure 5) and surface damage—specifically localized folding and delamination (Figure 4f,h). When subjected to cyclic loading, such damage gives rise to stress concentration and surface CRS relaxation, which thereby reduces the beneficial effects of SP treatment [5,30].

Hence, the key to optimizing the SP process lies in balancing the beneficial and detrimental factors. Expected improvement in fatigue performance can be achieved when beneficial factors dominate. Both the degree of work hardening (Figure 6) and the affected depth of CRS (Figure 7) were notably elevated for the 0.45 mmN specimen. However, it also exhibited excessively high surface roughness (Ra: 2.545 μm) along with severe surface damage (Figure 4g,h). For this reason, the fatigue performance of the 0.45 mmN specimen was worse than that of the 0.35 mmN specimen. Likewise, the 0.25 mmN specimen yielded better surface quality with less surface damage (Figure 4c,d and Figure 5), but was accompanied by weaker work hardening (Figure 6), lower surface CRS, and a thinner affected layer (Figure 7). Its fatigue performance was thus lower than that of the 0.35 mmN specimen. These results demonstrate an obvious phenomenon of SP treatment: an optimal fatigue strengthening effect cannot be achieved when the peening intensity is either too high or too low. To obtain the best fatigue performance, the SP process parameters, particularly for peening intensity, must be reasonably controlled to realize the synergistic optimization of various surface integrity factors.

Experimental date for the RSP specimen showed that an optimal balance of surface integrity factors was achieved under this condition. During the RSP treatment, the re-peening process effectively reduced surface damage by smoothing the initial peening indentations. This resulted in lowering the surface roughness (Ra) by 40.1%, while the detrimental effects were effectively mitigated. At the same time, the beneficial factors, including CRS, dislocation density, and work hardening, were further strengthened. Of all the peening conditions, thus, the RSP specimen thus exhibited the best fatigue performance (Figure 8).

**Figure 11:**
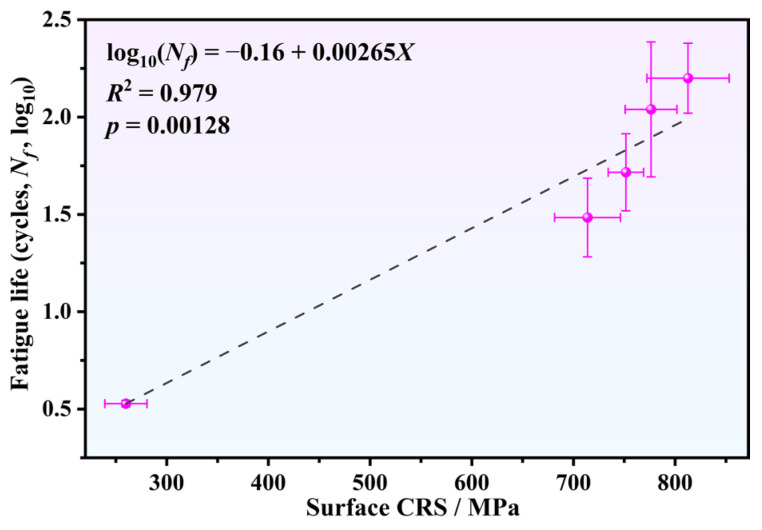
Semi-logarithmic linear regression analysis illustrating the dependence of fatigue life (maximum stress: 750 MPa) on the surface CRS. The dashed line represents the best linear fit.

A comparison of the data in Figure 7 and Figure 8 further reveals a distinct trend: higher surface CRS value corresponds to better fatigue performance. For the quantitative assessment of the relationship between surface CRS and fatigue life (maximum stress: 750 MPa), a semi-logarithmic regression analysis was conducted, which is illustrated in Figure 11. The experimental data in the plot (with error bars indicating scatter) exhibit a positive linear correlation between surface CRS and the logarithm of fatigue life (log_10_(*N_f_*) = −0.16 + 0.00265*X*, where *X* represents surface CRS). The statistical results (*R*^2^ = 0.979, *p* = 0.00128 < 0.01) further reveal that fatigue life increases exponentially with the increase in surface CRS. Although Figure 11 demonstrates a strong statistical correlation between surface CRS and fatigue life, the number of specimens used for this regression analysis is relatively limited. Experimental fatigue data inevitably exhibit statistical scatter, making it difficult to establish a causal relationship using regression analysis based on a limited dataset.

**Figure 12:**
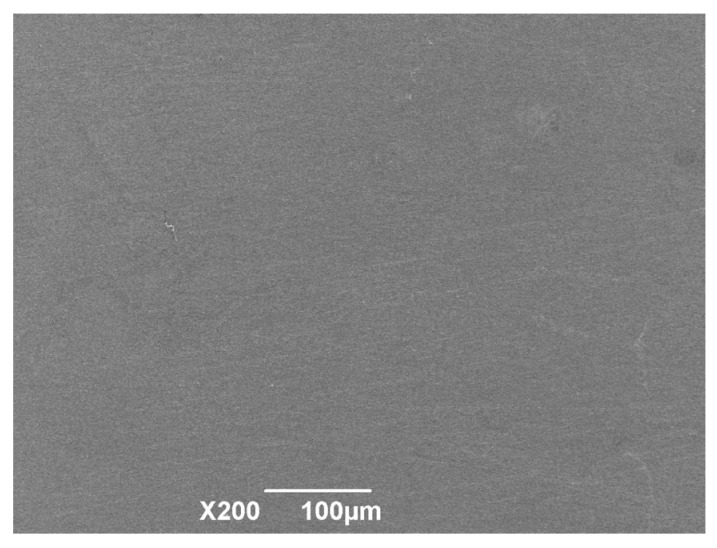
2D surface morphology of RSP + P specimen.

To overcome this shortcoming and clearly separate the effects of surface CRS and surface roughness on the fatigue performance, factor-separation experiments were performed. First, RSP specimens were cyclically loaded at 900 MPa for 30 cycles using a rotary bending apparatus. This pre-cycling relaxed surface CRS while maintaining surface topography. The resulting stress-relaxed specimens are denoted as RSP + R. Second, a separate set of RSP specimens was polished with 5000# water abrasive paper to a removal depth of roughly 20 μm. These polished specimens are denoted as RSP + P, and their 2D surface morphology is shown in Figure 12. The polishing treatment effectively smooths the surface. The surface roughness (Ra) of the RSP + P specimen was significantly reduced to a range of 0.169–0.245 μm, which is considerably lower than that of the BM specimen.

**Figure 13:**
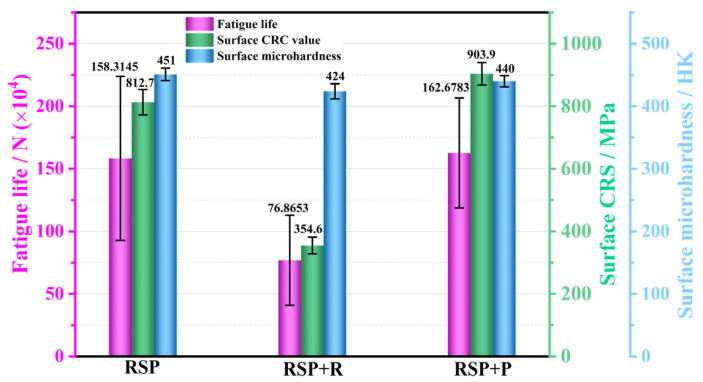
Fatigue life (maximum stress: 750 MPa), surface CRS, and surface microhardness of the RSP, RSP + R, and RSP + P specimens.

Figure 13 compares the fatigue life (maximum stress: 750 MPa), surface CRS, and surface microhardness among the RSP, RSP + R, and RSP + P specimens. As shown, the pre-cycling relaxation in the RSP + R specimens induced a substantial reduction in surface CRS from 812.7 MPa to 354.6 MPa, corresponding to a stress relaxation of 56.4%. Meanwhile, the surface microhardness decreased only slightly (from 451 HK to 424 HK), suggesting that the high-stress pre-cycling effectively relaxed the surface CRS while largely preserving the work-hardened state. Consequently, the RSP + R specimen experienced a marked 51.4% reduction in fatigue life (from 158.3145 × 10^4^ to 76.8653 × 10^4^ cycles). This indicates that the surface CRS value is critical to improving fatigue performance. These results agree well with what has been documented in previous works by various researchers [7,10,22]. For instance, Wang et al. [10] noted that the combined laser shock peening and SP treatment produced a marked improvement of about 35% in the high-cycle fatigue performance of Ti-6Al-4V alloy. The CRS with a higher surface value and greater affected depth was pivotal in improving the fatigue performance of this alloy. Paques et al. [22] also pointed out that CRS is the primary factor responsible for improved fatigue performance in Ti-6Al-4V, on condition that the cyclic stress stays below the yield strength. By eliminating the detrimental surface roughness (Figure 12) while retaining a high subsurface CRS (903.9 MPa) and microhardness (440 HK), the RSP + P specimens yielded a fatigue life of 162.6783 × 10^4^ cycles, which marginally exceeded that of the RSP condition (158.3145 × 10^4^ cycles). These results indicate that, although reducing surface roughness provides additional improvement in fatigue life, the magnitude of surface CRS plays a key role in the fatigue performance of TC17 titanium alloy.

## 5. Conclusions

In this study, TC17 titanium alloy specimens were prepared with SP and RSP treatments. The influence of surface integrity on fatigue performance was investigated comprehensively. The key findings can be organized below:

(1) High-density dislocations and pronounced work hardening were found in the surface layer for both SP- and RSP-treated specimens. High-intensity peening caused severe surface damage and led to the relaxation of the surface CRS. In contrast, the RSP treatment smoothed peening indentations formed by the initial peening, alleviated surface damage, raised surface CRS, and deepened the affected layer.
(2) The fatigue life for the 0.35 mmN specimen, under a maximum stress of 750 MPa, was remarkably enhanced by 31.4 times relative to the BM specimen. The RSP treatment further increased this ratio to 45.9. Meanwhile, the fatigue limit (at 10^7^ cycles) improved from 590 MPa for the BM specimen to 700 and 710 MPa for the 0.35 mmN and RSP specimens, respectively. Considering all these results, the RSP treatment achieved a better fatigue performance than the other conditions.
(3) Improving the fatigue performance via SP relies on achieving an appropriate balance of surface integrity, maximizing beneficial effects while reducing the accompanying detrimental ones. The CRS, dislocation multiplication, and work hardening act as beneficial factors, whereas surface roughness and surface damage serve as detrimental factors. By using two successive peening processes, the RSP treatment generates a synergistic optimization that preserves beneficial factors while keeping detrimental factors within acceptable limits. Then, the best fatigue performance was obtained for the RSP specimen.
(4) Factor-separation experiments on RSP + R and RSP + P specimens demonstrate that reducing surface roughness only brings a marginal enhancement in fatigue life, whereas the relaxation of surface CRS leads to a marked 51.4% reduction in fatigue life. Thus, among all beneficial factors, the magnitude of surface CRS is the key to improving fatigue performance of TC17 titanium alloy.

## Figures and Tables

**Table 1:** SP and RSP parameters of TC17 titanium alloy.

Peening Type	Almen Intensity/mmN	Coverage/%	Peening Angle/°	Specimen ID
S110	0.25	150	75~90	0.25 mmN
	0.35			0.35 mmN
	0.45			0.45 mmN
S110 + Z300	0.35 + 0.25			RSP

## Data Availability

The original contributions presented in this study are included in the article. Further inquiries can be directed to the corresponding author.
